# Analgesic strategies in the management of postoperative pain following uterine artery embolization for symptomatic fibroids: a systematic review

**DOI:** 10.1590/acb413626

**Published:** 2026-07-17

**Authors:** Manoela Massa Moura, Karen Ruggeri Saad, Edson Ferreira Gonçalves, Larissa Freire Golveia Silveira, Henrique Pereira Castro, Antônio Felipe, Paulo Fernandes Saad

**Affiliations:** 1 Universidade de Brasília – Faculty of Medicine – Medical course – Brasília (DF) – Brazil.; 2 Universidade de Brasília – Faculty of Medicine – Department of Morphology – Brasília (DF) – Brazil.; 3 Universidade de Brasília – University Hospital of Brasília – Brasília (DF) – Brazil.; 4 Universidade de Brasília – Faculty of Medicine – Department of Surgery – Brasília (DF) – Brazil.

**Keywords:** Uterine Artery Embolization, Leiomyoma, Postoperative Pain, Analgesia, Analgesics, Analgesics, Opioid

## Abstract

**Purpose:**

To identify and compare anesthetic and analgesic strategies employed during uterine artery embolization for symptomatic leiomyomas, assessing their effectiveness in reducing postoperative pain and opioid use and evaluating complication rates.

**Methods:**

A systematic review was conducted in accordance with the Preferred Reporting Items for Systematic reviews and Meta-Analyses guidelines. Searches were performed in PubMed, Embase, Web of Science, and Scientific Electronic Library Online. Randomized controlled trials and prospective cohort or case–control studies, published in the last 10 years, evaluating pain, opioid consumption, and/or complications, were included.

**Results:**

The total of 3,433 studies were screened, of which six met eligibility criteria. Superior hypogastric plexus block reduced pain and opioid use. Intra-arterial lidocaine decreased pain within 2–4 h and opioid consumption, with a transient effect; administration after vascular stasis was preferable. Controlled-release oxycodone reduced pain at 24 h and need for rescue medication without lowering mean consumption. Dexamethasone reduced pain at 12–24 h without impacting opioid use. No significant complications were reported.

**Conclusion:**

In the ischemic phase of post-uterine artery embolization pain, a superior hypogastric nerve block appears to provide the most consistent relief, with intra-arterial lidocaine representing an alternative when resources are limited. For the inflammatory phase, intravenous dexamethasone is the preferred option, whereas controlled-release oxycodone may be considered only in refractory cases.

## Introduction

Uterine leiomyomas are benign tumors resulting from abnormal proliferation of smooth muscle cells within the myometrium, classified according to location as subserosal, intramural, submucosal, or cervical. In general, only symptomatic fibroids are treated. Those with heavy menstrual bleeding, dysmenorrhea, or mass effect symptoms such as pelvic pain and pressure constitute the most common clinical presentations^1^. Therapeutic options for symptomatic fibroids include hysterectomy, myomectomy, endometrial ablation, and uterine artery embolization (UAE)^1^.

UAE is increasingly recommended due to shorter hospitalization, faster recovery, and lower complication rates when compared to hysterectomy—the former gold standard. Minimally invasive, the technique consists of occluding the arterial branches supplying fibroids, inducing ischemia and subsequent tumor necrosis^2^. The main limitation of UAE is the high incidence of severe pain, attributed primarily to transient myometrial ischemia after embolization. Typically, pain begins approximately 1 hour after the procedure, peaks between 5 and 7 hours, and progressively declines, enabling most patients to be discharged within 24 hours^3^.

The intensity of this pain is striking. In a cohort of 290 women, 35% reported pain of similar or greater magnitude than labor, despite non-steroidal anti-inflammatory drug (NSAID) administration and patient-controlled analgesia^4^. Moreover, post-embolization syndrome—characterized by diffuse abdominal pain, fever, nausea, vomiting, and leukocytosis—affects up to 40% of patients, further worsening postoperative recovery^5^. These findings highlight the need for multimodal analgesic regimens capable of attenuating pain while optimizing clinical outcomes.

Most analgesia protocols for UAE rely on NSAIDs combined with opioids, frequently via patient-controlled analgesia^6^. While effective, this strategy often requires high opioid doses, exposing patients to adverse events such as nausea, constipation, sedation, and respiratory depression. Beyond clinical implications, the increasing use and widespread availability of opioids have become a global concern, exemplified by the epidemic in the United States of America, where over 80,000 opioid-related overdose deaths were documented in 2023^7^.

Given these limitations, alternative strategies have been explored to reduce opioid exposure while maintaining analgesic efficacy. Invasive techniques, such as superior hypogastric nerve block (SHNB) and intra-arterial lidocaine injection, have demonstrated potential in alleviating ischemic pain and reducing narcotic use^6^. Meanwhile, systemic pharmacological adjuvants, such as corticosteroids and controlled-release opioids, have been tested as adjuncts to standard regimens.

Although preliminary findings are encouraging, no consensus has been reached on the optimal strategy for post-UAE pain management. The heterogeneity of protocols and outcomes underscores the need for a systematic analysis of the literature. Therefore, the aims of this review were to identify and compare anesthetic and analgesic strategies employed during UAE for symptomatic leiomyomas, assessing their effectiveness in reducing postoperative pain and opioid use in comparison to standard analgesia protocols. As a secondary objective, this review sought to evaluate complication rates in order to further delineate the safety profile and clinical applicability of these interventions.

## Methods

The present review was designed and reported following the Preferred Reporting Items for Systematic reviews and Meta-Analyses (PRISMA) 2020 recommendations^8^. The review process commenced with a structured literature search in PubMed, Embase, Web of Science, and Scientific Electronic Library Online (SciELO). The following descriptors were applied: *“uterine artery embolization” AND “anesthesia”; “embolização da artéria uterina” AND “anestesia”; “uterine artery embolization” AND “analgesia”; “embolização da artéria uterina” AND “analgesia”; “uterine artery embolization” AND “analgesics”; “embolização da artéria uterina” AND “analgésicos”; “uterine artery embolization” AND “postoperative analgesia”; “embolização arterial uterina” AND “analgesia pós-operatoria”; “uterine artery embolization” AND “complication”; “embolização da artéria uterina” AND “complicações”; “uterine artery embolization” AND “length of stay”; “embolização arterial uterina” AND “tempo de internação”; “uterine artery embolization” AND “pain”; “embolização arterial uterina” AND “dor”; “uterine artery embolization” AND “pain control”; and “embolização arterial uterina” AND “controle da dor”.*

Eligible studies included peer-reviewed randomized controlled trials and prospective cohort or case–control studies, published in Portuguese or English in the last ten years, that evaluated anesthesia type during UAE and reported on at least one relevant outcome—postoperative opioid use (timing and amount), complication rates, or pain control. Exclusion criteria comprised studies not directly addressing anesthesia in UAE, animal studies, and those with fewer than 10 participants per group.

**Figure 1:**
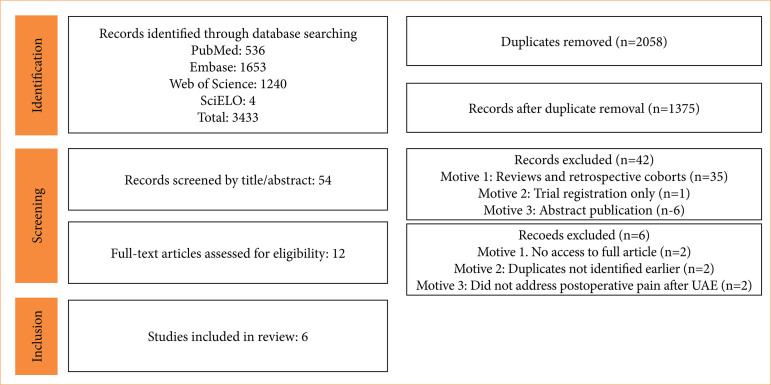
Strategy for literature search and study selection.

Source: Elaborated by the authors.

All potentially eligible articles were independently screened by two reviewers, and disagreements were resolved by a third. After duplicate removal, a two-stage selection process was conducted: an initial screening of titles and abstracts, followed by full-text assessment for eligibility. Inter-rater agreement was measured at each stage to ensure methodological consistency. The search and selection process is presented in the PRISMA flow diagram (Fig. 1). Studies meeting all predefined inclusion criteria were retained for analysis. Data extraction was performed by a single reviewer using a standardized form, capturing study characteristics, participant demographics, treatment details, and predefined outcomes of interest.

Methodological quality was assessed using the Cochrane Risk of Bias 2 (RoB2) tool for randomized controlled trials^9^ and the Newcastle–Ottawa Scale (NOS) for cohort studies^10^. Meta-analysis was not feasible due to study heterogeneity; therefore, results were synthesized narratively.

## Results

### Article selection

A total of 3,433 articles were initially identified. After duplicate removal and screening of titles and abstracts, 12 articles were retrieved for full-text review. Six studies met the inclusion criteria and were incorporated into the review. The selection process is detailed in the PRISMA flow diagram (Fig. 1). Methodological quality assessed using the RoB29 tool and the Newcastle–Ottawa Scale^10^, revealed low risk of bias, indicating the overall methodological quality of the included studies (Tables 1 and 2).

**Table 1:** Quality assessment of the case-control study using the Newcastle-Ottawa (NOS)<xref>^10^</xref>.

Author, year	Title	Study type	Participant selection	Comparability between groups	Outcome of interest	Final Score
Almazedi et al., 2014^11^	Impact of superior hypogastric nerve block during uterine fibroid embolisation on pain scores, opioid requirements, and same-day discharge: a case-control study	Case control	4/4	1/2	3/3	8/9 (high quality)

Source: Elaborated by the authors.

**Table 2:** Methodological assessment of randomized clinical trials using the Cochrane Risk of Bias 2 tool (RoB2)<xref>^9^</xref>.

Author, year	Title	Risk of bias due to the randomization process	Risk of bias due to deviations from intended interventions	Risk of bias due to missing outcome data	Risk of bias in outcome measurement	Risk of bias in selection of the reported result
Duvnjak and Andersen, 2020 ^3^	Intra-arterial lidocaine administration during uterine fibroid embolization to reduce the immediate postoperative pain: a prospective randomized study	Some concerns	Some concerns	Low	Low	Low
Noel-Lamy et al., 2017 ^4^	Intraarterial lidocaine for pain control in uterine artery embolization: a prospective, randomized study	Low	Some concerns	Low	Low	Low
Freire et al., 2017 ^12^	Controlled-release oxycodone improves pain management after uterine artery embolisation for symptomatic fibroids	Some concerns	Some concerns	Low	Low	Low
Kim et al., 2016 ^13^	The effects of single-dose dexamethasone on inflammatory response and pain after uterine artery embolisation for symptomatic fibroids or adenomyosis: a randomised controlled study	Low	Low	Low	Low	Low
Yoon et al., 2018 ^14^	Superior hypogastric nerve block as post-uterine artery embolization analgesia: a randomized and double-blind clinical trial	Low	Low	Low	Low	Low

Source: Elaborated by the authors.

### Anesthesic and analgesic protocols

In most studies included, standard analgesia consisted of opioids and NSAIDs, often combined with acetaminophen. Using conventional regimens as comparators, the studies evaluated distinct adjuvant interventions, grouped into two categories:

- Invasive approaches, such as SHNB and intra-arterial lidocaine administration;
- Conservative pharmacological strategies, such as controlled-release oxycodone and intravenous dexamethasone.

Complete protocols are summarized in Table 3.

**Table 3:** Analgesic protocols.

Author, year	Study design	Pain control				Drugs in the protocol		
		Control	Intervention	Pre-procedure		Peri-procedure	Post-procedure	Discharge
Duvnjak and Andersen, 2020 ^3^	Randomized clinical trial	Local anesthesia + opioids (including PCA) + NSAIDs	Local anesthesia + opioids (including PCA) + NSAIDs + intra-arterial lidocaine	Morphine 2 mg		Local anesthesia ± intra-arterial lidocaine 1% 10 mL (100 mg) in each uterine artery (total = 200 mg)	Morphine PCA (initial dose 2 mg + 1 mg bolus) + NSAIDs	Narcotics and NSAIDs as needed
Noel-Lamy et al., 2017 ^4^	Randomized clinical trial	Opioids + NSAIDs	Opioids + NSAIDs + intra-arterial lidocaine	Acetaminophen PO 1,000 mg + Ibuprofen PO 400 mg + Oxycodone PO 10 mg + Ondansetron IV 4 mg + Dexamethasone IV 6 mg		Midazolam + Fentanyl + Intra-arterial lidocaine 1% 100 mg	Acetaminophen PO 1,000 mg + Ibuprofen PO 400 mg + Oxycodone PO 5–15 mg (rescue) + Hydromorphone IV 0.5–1 mg (rescue)	Oxycodone PO + Acetaminophen (as needed)
Almazedi et al., 2014 ^11^	Case-control	Local anesthesia + opioids (including PCA) + NSAIDs + acetaminophen	Local anesthesia + opioids (including PCA) + NSAIDs + acetaminophen + SHNB	Acetaminophen 1 g + Ibuprofen CR 800 mg + Tramadol 100 mg + Oxycodone CR 5–10 mg		Local anesthesia ± SHNB; Fentanyl 25–200 µg IV + Dexamethasone 6.6 mg IV + Ondansetron 4 mg IV	Fentanyl 25–100 µg IV + Oxycodone 1–20 mg IV + Ondansetron 4 mg IV	Acetaminophen 1 g 6/6 h + Ibuprofen 400 mg 8/8 h + Morphine 10–20 mg 6/6h + Cyclizine 50 mg 6/6 h
Freire et al., 2017 ^12^	Randomized clinical trial	Spinal anesthesia + opioids (including PCA) + NSAIDs + metamizole	Spinal anesthesia + opioids (including PCA) + NSAIDs + controlled-release oxycodone	± Oxycodone CR 20 mg PO		Spinal anesthesia (bupivacaine 15 mg + intrathecal morphine 200 µg) + sedation with propofol	± Oxycodone CR 20 mg + Morphine PCA (2 mg/dose) + Ketoprofen IV 100 mg + Metamizole IV 2 g	± Oxycodone CR (up to 2nd postoperative day)
Kim et al., 2016 ^13^	Randomized clinical trial	Opioids (PCA) + NSAIDs	Opioids (PCA) + NSAIDs + IV dexamethasone	Ramosetron IV + Dexamethasone 10 mg IV		Fentanyl IV 50 µg + Ketorolac IV 60 mg	PCA IV (fentanyl 1,500 µg + ramosetron 0.3 mg/150 mL; basal 10 µg/h, bolus 20 µg) + Tramadol 75 mg + Acetaminophen 650 mg	ND
Yoon et al., 2018 ^14^	Randomized clinical trial	Local anesthesia + opioids (including PCA) + NSAIDs	Local anesthesia + opioids (including PCA) + NSAIDs + SHNB	Naproxen 500 mg PO, BID for 10 days (starting the night before procedure)		Lidocaine 1٪ with epinephrine (3 mL, dose-test) + SHNB with ropivacaine 0.5٪ (20 mL total) + Fentanyl + Midazolam IV	Naproxen 500 mg PO, Fentanyl IV (as needed), opioids IV as required (oxycodone, hydromorphone, meperidine), Antiemetics	Naproxen 500 mg PO; Acetaminophen or Codeine 30 mg or Acetaminophen + Tramadol (as needed)

PCA: patient-controlled analgesia; NSAIDs: nonsteroidal anti-inflammatory drugs; SHNB: superior hypogastric nerve block; CR: controlled release; PO: per os (oral); IV: intravenous; ND: no data. Source: Elaborated by the authors.

### Outcomes: pain, opioid consumption and complications

Key findings regarding study characteristics and clinical outcomes are presented in Table 4.

**Table 4:** Studies evaluating the impact of anesthesia type on opioid consumption, pain scores, and complication rates.

Author, year	Study design	N participants	Comparison	Opioid consumption	Pain scores	Complication rates
Duvnjak and Andersen, 2020 ^3^	Randomized clinical trial	36	Standard analgesia versus standard analgesia + intra-arterial lidocaine	IV morphine: 11.2 mg (lidocaine) *versus* 20.2 mg (control), *p* < 0.03	NRS 2 h: 42.7 ± 21.4 *versus* 61.1 ± 20.4, *p* < 0.04; NRS 4 h, 7 h, 10 h, 24 h: no significant difference	No lidocaine-related complications. Three patients (two intervention, one control) required re-hospitalization within three months; no adverse events reported
Noel-Lamy et al., 2017 ^4^	Randomized clinical trial	60	Standard analgesia versus standard analgesia + intra-arterial lidocaine (1%) mixed with embolic particles (A) or injected after vascular stasis (B)	In-hospital morphine: 8.5 (A) *versus* 11.1 (B) *versus* 17.4 mg (control), *p* < 0.05; post-discharge morphine: 11.1 (A) *versus* 16.3 (B) *versus* 21 mg (control), *p* < 0.05	VAS 4 h: 28.6 ± 24.5 (A) *versus* 35.8 ± 22.6 (B) *versus* 59.4 ± 30.3 (control), *p* = 0.001; VAS 7 and 24 h: no significant differences	No major adverse events. Group A (lidocaine mixed with particles) showed higher rates of incomplete fibroid necrosis (38.9 *versus* 77.8 and 75% in groups B and control, respectively; *p* = 0.045)
Almazedi et al., 2014 ^11^	Case-control	119	Standard systemic analgesia versus standard systemic analgesia + SHNB	IV morphine: 16 mg (SHNB) *versus* 22 mg (control), *p* = 0.004; oral equivalent (day-case): 44 mg (SHNB) *versus* 80 mg (control), *p* = 0.020; IV fentanyl: 50 μg (SHNB) *versus* 125 μg (control), *p* = 0.015	Mean NRS 0–6 h: 2.6 *versus* 3.8, *p* = 0.031; day-case: 2.7 *versus* 4.6, *p* = 0.032	No SHNB-related complications
Freire et al., 2017 ^12^	Randomized clinical trial	60	Analgesia protocol with spinal anesthesia versus standard protocol (with spinal anesthesia) + controlled-release oxycodone	Morphine at 24 h: 8 mg (CRO) *versus* 16 mg (control), *p* = 0.130; ٪ r equiring rescue morphine: 46.7٪ (CRO) *versus* 76.7٪ (control), *p* = 0.017	No difference at 2 h, 6 h, 12 h (*p* > 0.05); lower at 24 h in CRO group (~0 *versus* ~3), *p* = 0.029	No major complications; fewer cases of nausea in CRO group (20 *versus* 46.7%, *p* = 0.029)
Kim et al., 2016 ^13^	Randomized clinical trial	59	Standard analgesia + placebo versus standard analgesia + IV dexamethasone	IV fentanyl: 822 ± 296 μg (dexa) *versus* 945 ± 426 μg (placebo), *p* = 0.206	No difference at 6 h; significant reduction with dexamethasone at 12 and 24 h (*p* < 0.05); numerical values not reported	No dexamethasone-related complications; safety profile comparable to placebo
Yoon et al., 2018 ^14^	Randomized clinical trial	44	Standard analgesia + sham subcutaneous lidocaine infiltration + simulated maneuvers versus standard analgesia + SHNB	Morphine equivalent in PACU: 5.1 ± 5.8 mg (SHNB) *versus* 11 ± 9 mg (sham), *p* = 0.014; IV fentanyl: 56 ± 67 μg (SHNB) *versus* 124 ± 91 μg (control), *p* = 0.009	VAS immediately post-UAE: 1.1 ± 2.1 *versus* 2.6 ± 2.8, *p* = 0.026; no significant differences thereafter	No major SHNB-related complications. Minor events: low back pain (6), difficulty advancing the needle (1), transient tachycardia (1), mild bleeding (1), transient foot numbness (1).

SHNB: superior hypogastric nerve block; IV: intravenous; NRS: numeric rating scale; CRO: controlled-release oxycodone; VAS: visual analog scale; PACU: post-anesthesia care unit; UAE: uterine artery embolization. Source: Elaborated by the authors.

Among studies that assessed SHNB, Almazedi et al.^11^ reported significant reduction in pain up to 6 h post procedure (mean numeric rating scale 0–6 h = 2.6 versus 3.8; *p* = 0.031), whereas Yoon et al.^14^ found differences only in the immediate postoperative period (visual analog scale = 1.0 ± 2.1 *versus* 2.6 ± 2.0; *p* = 0.01). Both studies demonstrated lower opioid consumption in the intervention groups: IV morphine (16 *versus* 22 mg; *p* = 0.004), oral morphine equivalents during outpatient management (44 versus 80 mg; *p* = 0.020), and IV fentanyl (50 *versus* 125 µg; *p* = 0.015) in Almazedi et al.^11^; and oral morphine equivalents (5.1 ± 5.8 *versus* 11.0 ± 9.0 mg; *p* = 0.014) and IV fentanyl (56 ± 67 *versus* 124 ± 91 µg; *p* = 0.009) in Yoon et al.^14^. Reported complications were limited to minor, self-limiting events such as low back pain (n = 6), tachycardia (n = 1), and transient paresthesia.

Regarding intra-arterial lidocaine, Duvnjak and Andersen3 found significant pain reduction only at 2 h post-procedure (42.7 ± 21.4 *versus* 61.1 ± 20.4; *p* < 0.02), with no differences at later assessments. Noel-Lamy et al.4 compared three groups: lidocaine mixed with embolic particles, lidocaine administered after vascular stasis, and control. At 4 h, pain scores were significantly lower in both intervention groups compared with control (28.6 ± 24.5; 35.8 ± 22.6; 59.4 ± 30.3; *p* = 0.001), but no differences were observed at 7 or 24 h. Opioid consumption was also reduced in the intervention groups, both during hospitalization (8.5 *versus* 11.1 *versus* 17.4 mg; p < 0.05) and after discharge (11.1 *versus* 16.3 *versus* 21.0 mg; *p* < 0.05). However, Noel-Lamy et al.4 reported a higher rate of incomplete fibroid necrosis in the first group (38.9 *versus* 77.8 *versus* 75%; *p* = 0.045). No other adverse events were identified.

As for conservative pharmacological strategies, Freire et al.^12^ observed significant pain reduction only at 24 h (numeric rating scale ≈ 0 *versus* ≈ 3; *p* = 0.029), with no differences at 2, 6, or 12 h post-procedure (*p* > 0.05). Mean morphine consumption did not differ between groups (8 *versus* 16 mg; *p* = 0.130), although fewer patients in the oxycodone group required additional rescue morphine (46.7 *versus* 76.7%; *p* = 0.017). Kim et al.^13^ reported significant pain reduction with dexamethasone at 12 and 24 h (*p* < 0.05), but not in the first 12 h. Fentanyl consumption was similar between groups (822 ± 296 *versus* 945 ± 426 µg; *p* = 0.206). None of the studies reported complications attributable to these interventions.

## Discussion

Uterine artery embolozation has become established as a minimally invasive alternative for the management of symptomatic leiomyomas, offering faster recovery and lower complication rates compared with hysterectomy^1^. Its main limitation, however, is postoperative pain, which is often severe in the first hours after the procedure. Pain is the most frequently reported symptom following UAE, affecting approximately 90% of patients^15^. This highlights the magnitude of the analgesic challenge and reinforces the need for adjuvant strategies to optimize standard protocols.

### Postoperative pain

Studies on SHNB consistently demonstrated immediate pain relief, but results diverged regarding the duration of effect. Almazedi et al.^11^ observed benefits lasting up to 6 h, whereas Yoon et al.^14^ found differences only in the immediate postoperative period. This discrepancy may reflect both methodological differences and the natural evolution of pain pathophysiology, which is predominantly ischemic in the first hours and shifts to an inflammatory profile after 6–12 h^3^. Thus, SHNB appears most effective in the initial phase, without sustained impact later.

Intra-arterial lidocaine also significantly reduced early pain, though its effect was limited by the drug’s short half-life. Duvnjak and Andersen^3^ reported benefit at 2 h, and Noel-Lamy et al.^4^ at 4 h, with no subsequent differences. These findings align with the meta-analysis by Liu and Li^16^, which demonstrated pain reduction up to 4 h post-UAE. In Noel-Lamy’s study^4^, administration mixed with embolic particles resulted in lower pain scores but at the cost of a higher rate of incomplete fibroid necrosis, possibly due to distal vasospasm. Accordingly, administration after vascular stasis seems preferable, as it provides analgesia without compromising embolization efficacy.

Pharmacological adjuvants exhibited more delayed effects. Controlled-release oxycodone reduced pain only at 24 h, while intravenous dexamethasone showed benefit between 12–24 h, without immediate effect. These findings suggest that such interventions primarily target the inflammatory component of pain, reinforcing their role as complements rather than substitutes for early pain-control strategies.

### Opioid consumption

Patients treated with SHNB or intra-arterial lidocaine showed consistent reductions in opioid requirements, thereby reducing exposure to opioid-related adverse effects. Opioid consumption can also serve as an indirect marker of pain intensity. From this perspective, the results of Yoon et al.^14^ become more comprehensible: the absence of significant differences in pain scores after the immediate period may be attributed to greater opioid use in the control group, which likely masked perceived pain and leveled scores between groups. With respect to pharmacological adjuvants, oxycodone reduced the proportion of patients requiring rescue morphine but not mean consumption, while dexamethasone showed no significant effect on this outcome.

### Complications

No study reported major complications. SHNB was associated only with mild, self-limiting events (low back pain, paresthesia, tachycardia). Duvnjak and Andersen3 found no adverse events with intra-arterial lidocaine, whereas Noel-Lamy et al.4 identified a higher rate of incomplete fibroid necrosis in the group receiving lidocaine mixed with embolic particles. In studies evaluating oxycodone and dexamethasone, no attributable adverse events were reported, with a safety profile comparable to control groups.

### Synthesis of findings and limitations

Overall, SHNB consistently reduced immediate pain and opioid consumption, though with short-term benefit and dependence on technical expertise. Intra-arterial lidocaine also reduced pain and opioid use during the early postoperative period, particularly when administered after vascular stasis, but its effect was transient. Pharmacological adjuvants such as oxycodone and dexamethasone acted mainly during the later inflammatory phase, with modest impact on analgesia and opioid reduction.

Taken together, these findings suggest that different strategies complement each other over the course of pain evolution, reinforcing the rationale for multimodal protocols. Nonetheless, interpretation must consider important limitations, including the small sample sizes, heterogeneity of analgesic regimens, and short follow-up duration. Additional concerns include difficulties with blinding and the lack of standardized pain assessment tools and time points, which hinder comparability across studies. Furthermore, the absence of cost-effectiveness analyses is noteworthy, as such evaluations are essential to determine the feasibility of integrating these strategies into clinical practice.

## Conclusion

Postoperative pain after UAE is best managed with multimodal protocols tailored to the temporal dynamics of pain, addressing both the early ischemic and the later inflammatory phases. Evidence indicates that SHNB provides the most consistent and sustained relief in the immediate postoperative phase, whereas intra-arterial lidocaine—when administered after vascular stasis—offers a technically simpler approach, while only transiently effective, and may serve as an alternative when SHNB is not feasible.

For the inflammatory phase, intravenous dexamethasone should be prioritized, given its earlier onset and consistent efficacy at 12–24 h. By contrast, controlled-release oxycodone provides a delayed effect, observed at 24 h, and, as an additional opioid, holds limited value in an opioid-sparing context; its use should therefore be restricted to refractory cases.

Accordingly, the optimal regimen combines SHNB (or intra-arterial lidocaine as an alternative) with intravenous dexamethasone, integrated into standard care with NSAIDs and patient-controlled opioids. Future multicenter, standardized trials with extended follow-up and cost-effectiveness analyses are essential to consolidate these recommendations and support their incorporation into routine clinical practice.

## Data Availability

All data sets were generated or analyzed in the current study.
